# Stress among Parents of Children with and without Autism Spectrum Disorder: A Comparison Involving Physiological Indicators and Parent Self-Reports

**DOI:** 10.1007/s10882-017-9547-z

**Published:** 2017-03-31

**Authors:** Ciara Padden, Jack E. James

**Affiliations:** 1 Tizard Centre, Cornwallis North East, University of Kent, Canterbury, Kent, CT2 7NF UK; 2 School of Psychology, National University of Ireland, Galway, Ireland; 3 Department of Psychology, Reykjavik University, Reykjavik, Iceland

**Keywords:** Autism spectrum disorder, Parenting stress, Cortisol, Alpha-amylase, Ambulatory blood pressure

## Abstract

Parents of children with Autism Spectrum Disorder (ASD) have been reported as experiencing higher levels of stress and poorer physical health than parents of typically developing children. However, most of the relevant literature has been based on parental self-reports of stress and health. While research on physiological outcomes has grown in recent years, gaps still exist in our understanding of the physiological effects, if any, of stress related to parenting a child with ASD. The present study compared parent-reported stress, anxiety, and depression, as well as selected physiological measures of stress (i.e., cortisol, alpha-amylase, and ambulatory blood pressure and heart rate) between matched groups of parents of children with (*N =* 38) and without (*N* = 38) ASD. Participants completed questionnaires, collected saliva samples for the purpose of measuring cortisol and alpha-amylase, and wore an ambulatory blood pressure monitor for 24 h. Parents of children with ASD reported significantly higher levels of parental distress, anxiety, and depression than parents of typically developing children. Parent-reported distress, anxiety, depression, and health were not correlated with physiological measures. With the exception that parents of children with ASD had significantly lower cortisol levels 30 min after waking, no other significant group differences were found for physiological measures. Parents of children with ASD reported significantly higher use of a number of adaptive coping strategies (e.g., emotional support) in comparison to parents of typically developing children. Results are discussed in the context of implications for future research directions, stress research, and practical implications for parental support.

Parenting is inherently challenging, even in relation to normative events experienced by every parent (Glasberg et al. 2006), and potentially more so for parents of children with additional needs. Previous research has consistently reported higher levels of parenting stress among parents of children with Autism Spectrum Disorder (ASD) than parents of typically developing children and children with other disabilities (Hayes and Watson 2013). Given that chronic stress can have adverse effects on a range of physical as well as psychological outcomes (Loft et al. 2007), it is perhaps not surprising that parents of children with ASD have also been found to report poorer health and more illnesses than parents of children with typical development (e.g., Allik et al. 2006; Smith et al. 2012) and children with other disabilities (e.g., Mugno et al. 2007). However, most studies of stress and health in parents of children with ASD have relied on parental self-reports of stress that are subject to potential bias (Romanczyk and Gillis 2006) and provide only limited information about underlying mechanisms of action.

Research into stress-related physiological processes associated with caring for a child with ASD began only relatively recently (e.g., Gallagher and Whiteley 2012; Marinovic-Curin et al. 2008). One of the main avenues of research has been the investigation of salivary biomarkers. In particular, salivary cortisol and, to a lesser extent, salivary alpha-amylase (sAA) have been used to measure hypothalamic-pituitary-adrenal (HPA) axis and sympathetic nervous system (SNS) activity, respectively (e.g., Adam et al. 2006; Granger et al. 2007). Dysregulation of these systems has been associated with health issues (e.g., Adam and Kumari 2009; Clow et al. 2004; Granger et al. 2007).

However, results from studies comparing cortisol levels between parents of children with and without ASD have been mixed. Marinovic-Curin et al. (2008) found that cortisol levels did not differ significantly between families of children with ASD and those of children with typical development. Conversely, after adjusting for negative affect, Ruiz-Robledillo and Moya-Albiol (2013) found that parents of children with Asperger syndrome had significantly larger morning cortisol responses than parents of typically developing children. Seltzer et al. (2010), on the other hand, found that mothers of adolescents and adults with ASD had significantly *lower* morning cortisol responses than mothers of typically developing adolescents and adults. Lower morning and daily cortisol levels, in addition to lower cortisol responses to acute stress, have subsequently been reported in a number of studies (e.g., De Andres-Garcia et al. 2012; Lovell et al. 2015; Ruiz-Robledillo et al. 2013). These findings suggest that many parents of children with ASD may be at risk of blunted cortisol responses.

Although a larger cortisol response is often associated with *acute* stress, blunted cortisol responses has been found to be associated with a history of *chronic* or prolonged exposure to stress (Adam et al. 2006). Blunted cortisol activity has been hypothesised to develop as a protective response among individuals who experience chronic stress, while in turn producing side-effects including symptoms of pain, fatigue, high stress sensitivity, and decreased immunity (Fries et al. 2005). Low cortisol levels have also been reported among individuals with post-traumatic stress disorder (PTSD), while there is some evidence of elevated SNS activity in this population (i.e., augmented norepinephrine levels in cerebrospinal fluid, and higher plasma catecholamine levels; Fries et al. 2005). Given that the stress responses of some parents of children with ASD have been compared to symptoms of PTSD (Casey et al. 2012), there is a case to be made for exploring both HPA-axis and SNS activity among parents of children with ASD to determine if there are similarities to previous findings for individuals with PTSD. While little is known about sAA levels among parents of children with ASD, the two studies that have been conducted to date (Foody et al. 2014, 2015) reported slightly elevated levels of sAA among parents of children with ASD. However, neither study included a control group of parents of typically developing children. Thus, further investigation of both cortisol and sAA levels among parents of children with ASD compared to a control group could provide useful information about both HPA-axis and SNS activity in this population.

Similarly, there is little known about the effects of caring for a child with ASD on cardiovascular (CV) activity. CV activity is of particular health concern given that stress is a significant risk factor for hypertension, which is the single most important risk factor for stroke and an important predictor of CV disease in general (World Heart Federation 2013). One feature of the large body of CV research is the emphasis that has been placed on the use of laboratory-based methods for inducing *reactivity* to psychological stress (Turner 1994). However, the contrived nature of such studies may not be representative of how people typically respond to stressors encountered in daily life (Turner 1994). Ambulatory blood pressure (ABP) monitoring, which involves repeated observations of blood pressure (BP) and heart rate (HR) under natural conditions, provides opportunities for measuring the effects of real-life stressors in the natural environment. ABP monitoring has been reported to be superior to office-based BP in predicting CV mortality (Dolan et al. 2005). Additionally, it is the only non-invasive technique for measuring night-time BP.

To our knowledge, ambulatory blood pressure in parents of children with ASD has only been investigated in three studies (Foody et al. 2014, 2015; Gallagher and Whiteley 2012), of which only one included a control group of parents with typically developing children (Gallagher and Whiteley 2012). Gallagher and Whiteley (2012) reported that parents of children with developmental disabilities (including ASD) had higher daily BP than control parents. However, only 37% of the total sample were parents of children with ASD, and it was not possible to separate their data from parents of children with other developmental disabilities. Furthermore, only 16% of the sample were fathers. Consequently, knowledge about the respective cardiovascular effects of caring for a child with ASD on mothers and fathers remains limited.

Gallagher and Whiteley (2012) also observed an association between social support and daytime systolic BP, wherein social support appeared to buffer the impact of stress on BP. Previous parent-report studies found lower levels of social support predicted higher levels of stress, depression, and anxiety among parents of children with ASD (Boyd 2002). Similarly, there is evidence that use of different coping strategies may have potential to alleviate or exacerbate stress levels among this population. For instance, Hastings et al. (2005) found that positive coping and reframing were associated with lower levels of depression in mothers and fathers of children with autism, while both Hastings et al. (2005) and Dunn et al. (2001) found that escape-avoidance was related to a higher number of negative psychological outcomes. Factors such as mindfulness and self-esteem have also been found to predict cortisol levels among parents of children with ASD (Dykens and Lambert 2013; Lovell et al. 2012). However, relatively little is known about potentially protective factors in the context of the physiological impact of chronic stress on parents of children with ASD. Thus, the role of factors such as social support and coping strategies should be further evaluated in studies of physiological outcomes within this population.

Thus, although it has been established that parents of children with ASD tend to report significantly higher levels of stress than parents of children with typical development or other disabilities, little is known about the physiological impact of stress related to parenting a child with ASD. In the present study, we examined the physiological effects of stress related to caring for a child with ASD, with the aim of addressing gaps in the existing literature. The primary aim was to compare physiological markers of stress between parents of children with and without ASD. The secondary aim was to explore potential differences between males and females, with the inclusion of matched parent dyads ensuring equal representation of males and females, which is important given that fathers are underrepresented in the existing literature. These comparisons were conducted as follows:

1. We explored cortisol levels, since mixed findings have been reported to date. More specifically, in light of some evidence of a pattern of lower cortisol levels among parents of children with ASD (e.g., Ruiz-Robledillo et al. 2013; Seltzer et al. 2010), we hypothesised that parents of children with ASD would display blunted morning cortisol responses compared to parents of typically developing children. It was hypothesised that this would be consistent for males and females given that previous comparisons of cortisol between mothers and fathers of children with ASD did not identify significant gender differences (Foody et al. 2015).
2. To determine whether there is support for a similar profile to that found for some adults with PTSD (i.e., blunted cortisol and elevated sAA levels), sAA was also measured, with an expectation that parents of children with ASD would have significantly higher levels of sAA than parents of typically developing children. Based on previous research that identified similar levels of sAA among mothers and fathers of children with ASD (Foody et al. 2015), it was expected that similar findings would be observed for males and females in both groups.
3. We also aimed to further understand CV activity in parents of children with ASD, hypothesising that they would have higher ABP than parents of typically developing children when compared. It was further hypothesised that males would present with higher BP and lower HR than females, given known gender differences in BP and HR (Fuster and Kelly 2010).

Although the majority of literature within the field focuses on parental reports of health and wellbeing, it is unclear whether such self-reports are reliable indicators of physiological activity. Accordingly, our third aim was to explore whether parent-report measures would correlate with physiological measures, making no specific prediction regarding these correlations. Finally, we aimed to compare levels of social support and coping strategies between the two parenting groups, given that relatively little is known about these variables in the context of physiological outcomes for parents of children with ASD.

## Methods

### Participants

Two groups of parents participated in this study, with a total sample of 76 parents. Participants from both groups were recruited through mainstream or special education schools, parent groups, or from the community using public announcements, and did not receive any payment. The ASD group (*N* = 38) comprised 19 mother-father dyads who had a child with ASD. Children were required to have received an independent diagnosis from a qualified clinician in order to be included in the study. Diagnoses had been received prior to the introduction of the DSM-5 (American Psychiatric Association 2003), therefore DSM-IV-TR (American Psychiatric Association 2000) diagnostic categories were included (i.e., a formal, independent diagnosis of any ASD, including autism, Asperger syndrome or pervasive developmental disorder not otherwise specified; PDD-NOS). Furthermore, parents completed the Gilliam Autism Rating Scale (GARS-2; Gilliam 2006), a norm-referenced assessment tool to assess severity of ASD symptoms (see Table 1). All total and subscale scores fell within the ‘very likely’ probability of autism category (i.e., Autism Index score ≥ 85 or subscale score ≥ 7; Gilliam 2006), providing support for the independent diagnoses that children had received.

**Table 1:** Mean scores for child behaviour problems and adaptive behaviour in the ASD (*N* = 19) and control (*N* = 19) groups

Measure	ASD group		Control group	
	M *(SD)*	Range	M *(SD)*	Range
GARS^a^: Stereotyped behaviour	10.42 (2.55)	7–15	2.26 (1.63)	1–5
GARS^a^: Communication	10.37 (3.39)	3–16	2.11 (1.82)	1–6
GARS^a^: Social interaction	10.74 (1.70)	7–13	1.79 (1.27)	1–5
GARS^a^: Autism index	103.32 (10.52)	83–126	46.68 (12.41)	14–68
BPI^b^: SIB frequency	7.26 (5.34)	0–19	0.79 (1.93)	0–7
BPI^b^: SIB severity	4.74 (3.38)	0–12	0.58 (1.30)	0–4
BPI^b^: Stereotypy frequency	27.68 (19.40)	2–68	1.37 (2.83)	0–10
BPI^b^: Stereotypy severity	16.05 (10.37)	2–42	0.84 (1.68)	0–5
BPI^b^: Aggression frequency	5.74 (5.15)	0–18	1.11 (2.45)	0–9
BPI^b^: Aggression severity	3.95 (3.67)	0–15	0.74 (1.48)	0–4
Vineland^c^: Communication domain	64.37 (14.85)	40–91	119.89 (15.97)	89–149
Vineland^c^: Daily living domain	65.32 (12.85)	43–93	113.37 (16.46)	95–155
Vineland^c^: Socialisation domain	62.74 (12.48)	38–85	119.37 (18.94)	96–160
Vineland^c^: Motor domain	73.82 (15.20)	56–102	107.09 (11.68)	84–124
Vineland^c^: Composite score	63.11 (11.86)	38–88	119.84 (17.65)	100–160

^**a**^GARS: Gilliam Autism Rating Scale (Gilliam 2006).

^b^BPI: Behavior Problems Inventory (Rojahn et al. 2001).

^c^Vineland: Vineland Adaptive Behavior Scales (Sparrow et al. 2005).

Mean age was 41.05 years (*SD =* 4.55) for mothers and 42.16 years (*SD =* 5.46) for fathers. Eleven percent of mothers and 79% of fathers were in full-time employment. On average, it was 4 years since the child had been diagnosed with ASD (*M* = 3.67, *SD* = 3.01, range: 0.25–11.33 years). Children in the ASD group had a mean age of 7.34 years (*SD* = 3.40), and the majority (79%) were boys. Autism was reported as the primary diagnosis for 16 (84%) of the children, with two (11%) reported as having PDD-NOS and one reported as having Asperger syndrome. Children in the ASD group had an average of 2.58 diagnoses (*SD =* 1.17), with comorbid diagnoses including mild (26%), moderate (16%), and severe (11%) intellectual disability (ID), attention-deficit hyperactivity disorder (ADHD; 11%), epilepsy (26%), physical disability (11%), and gastrointestinal issues (42%). Data on child behaviour problems and adaptive behaviour are reported in Table 1. These data were collected using the Behaviour Problems Inventory (BPI-01; Rojahn et al. 2001) and the Vineland Adaptive Behavior Scales (Vineland-II; Sparrow et al. 2005).

Parents were recruited to the control group if they had a typically developing child of the same gender and aged within 18 months of the matched child with ASD. To be included in the study, the child must never have had an assessment referral, nor be in receipt of a diagnosis of any intellectual, developmental, or physical disability, or any chronic illness. Furthermore, parents completed the GARS-2 (Gilliam 2006), with all mean scores falling within the ‘unlikely probability’ of autism category (i.e., Autism Index score ≤ 69 or subscale score of 1–3; Gilliam 2006). Parents in the control group were also required to be aged within 10 years of the matched parent from the ASD group. The matched control group (*N* = 38) included 19 mother-father dyads. Mean age was 36.94 (*SD* = 4.53) for mothers and 38.83 (*SD* = 5.66) for fathers. Twenty-six percent of mothers and 79% of fathers were in full-time employment. As with the ASD group, 79% of the children in the control group were boys, with a mean age of 6.79 years (*SD* = 3.29). Children in the control group did not have any reported diagnoses of any psychological or medical conditions.

### Psychometric Measures

Participants completed a questionnaire booklet, which included inventories that measured parent-reported stress, anxiety, and depression, in addition to certain child and parent variables. These measures are described below.

#### Parenting Stress Index-Short Form (PSI-SF)

The PSI-SF (Abidin 1995) is a 36-item self-report instrument that measures perceived stress related to the role of parenting. It contains statements related to parenting such as, “I feel trapped by my responsibilities as a parent”, and individuals rate their level of agreement with each statement on a 5-point Likert scale from 1 (*strongly agree*) to 5 (*strongly disagree*). Good internal consistency, test-retest reliability, and validity have been demonstrated (Abidin 1995). The present study used only the parental distress subscale, which has been reported to be the only subscale of the PSI-SF valid for use with parents of children with ASD (Zaidman-Zait et al. 2010). Strong internal reliability was found for the parental distress subscale (Cronbach’s α = .91).

#### Hospital Anxiety and Depression Scale (HADS)

The HADS (Zigmond and Snaith 1983) is a self-report instrument that includes two 7-item subscales to measure perceived anxiety and depression without contamination by reports of physical symptoms. Each item is rated on a 4-point Likert scale ranging from 0 to 3, with response options differing for each item. The scores for all seven items that make up the anxiety and depression subscales were then added to create the anxiety and depression subscales. High internal reliability and validity have been reported (Moorey et al. 1991; Zigmond and Snaith 1983). In the present study, good internal reliability was found for both anxiety (α = 85) and depression (α = .80) subscales.

#### Parental Responsibility Scale (PRS)

The PRS (McBride and Mills 1993) is a 14-item measure of perceived parenting responsibility. Common childcare tasks are listed (e.g., “Spending special time with the child at bedtime”). Respondents designate which parent has the primary responsibility for each task using a 5-point Likert scale ranging from 1 (*almost always completed by me*) to 5 (*almost always completed by my partner*). All 14 items were then combined to create the total PRS score. Moderate internal consistency, test-retest reliability, and validity have been reported (McBride and Mills 1993). Strong internal reliability was found in the present study (α = .92).

#### Brief COPE

The Brief COPE (Carver 1997) is a 28-item self-report measure of coping strategies. It is an abbreviated version of the COPE Inventory (Carver et al. 1989). It includes 14 scales, with two items per scale. Scales retained from the COPE Inventory are self-distraction, active coping, denial, substance use, use of emotional support, use of instrumental support, behavioural disengagement, venting, positive reframing, planning, humour, acceptance, and religion, with a self-blame scale added. Respondents rate statements about different coping strategies on a 4-point Likert scale ranging from 1 (*I haven’t been doing this at all*) to 4 (*I’ve been doing this a lot*). The two items from each subscale were then combined to produce the 14 subscale scores. The factor structure was found to be consistent with the COPE Inventory, and good internal reliability has been reported (Carver 1997). In the present study, self-distraction and venting were removed due to poor internal reliability (α < .60), while acceptable to strong internal reliability was found for the remaining subscales (α = .72–.91).

#### Short Form Social Support Questionnaire (SSQ)

The SSQ (Sarason et al. 1987) is a measure of perceived social support, which assesses both social support quantity and quality. It is an abbreviated version of the original 27-item Social Support Questionnaire (Sarason et al. 1983). Respondents are presented with six statements and are asked to list up to nine individuals who provide this type of support to them. These scores are added to provide the quantity subscale. They then rate their level of satisfaction with this type of support using a 6-point Likert scale ranging from 0 (*very dissatisfied*) to 6 (*very satisfied*), which are added to produce the quality subscale. Both subscales have been found to have high internal consistency and test-retest reliability, and strong factorial validity has also been reported (Sarason et al. 1987). In the present study, strong internal reliability was found (α = .84–.92).

#### Pittsburgh Sleep Quality Index (PSQI)

The PSQI (Buysse et al. 1989) is a 19-item scale that assesses sleep quality and disturbances over a 1-month period. It generates a global score and seven component subscales: subjective sleep quality, sleep latency, sleep duration, sleep disturbances, habitual sleep efficiency, use of sleeping medication, and daytime dysfunction. Only the global score was used in the present study. This is a total score that is created by combining all seven component subscale scores. Acceptable internal consistency, test-retest reliability, and validity have been reported (Buysse et al. 1989), while acceptable internal reliability was also found in the present study (α = .77).

### Demographic and Health Questionnaires

The questionnaire booklet included questions about participant age, employment status, and supports received. Health-related information was also collected, including smoking status, activity levels, caffeine intake, illnesses, and medication usage. In addition, information was collected on child and family characteristics, including child age, gender, and diagnostic information.

### Saliva Collection

The use of salivary cortisol as a marker of HPA-axis activity has been widely documented (e.g., Adam and Kumari 2009; Clow et al. 2004; Pruessner et al. 2003), and the use of sAA as a marker of SNS activity has also been reported (e.g., Granger et al. 2007; Rohleder and Nater 2009). In the present study, saliva samples were collected from under the front of the tongue using Salimetrics Oral Swabs (SOS; Salimetrics Europe, Suffolk, UK). The SOS is a small polymer swab that absorbs saliva from the mouth (Salimetrics 2012b). Participants were advised not to schedule data collection for at least 2 days after any dental work. Participants were also advised to avoid alcohol for 24 h before collecting saliva, and to avoid tooth-brushing, smoking, eating, drinking, and exercising in the 60 min before collecting saliva samples.

### Cardiovascular (CV) Assessment

CV measures were taken using an Oscar 2™ ambulatory blood pressure (ABP) monitor with an Orbit™ cuff (SunTech Medical Ltd., Oxfordshire, England). The reliability and validity of this monitor have been demonstrated (Goodwin et al. 2007). The cuff was programmed to inflate every 20 min between 8:00 and 22:00 (“daytime”), and every 45 min between 22:00 and 8:00 (“night-time”). Systolic blood pressure (SBP), diastolic blood pressure (DBP), and heart rate (HR) were recorded for each reading. CV data were downloaded from the monitor using the SunTech AccuWin Pro v3 ABPM software (SunTech Medical Ltd., Oxfordshire, England). Participants were provided with a print-out of the ABP data. In the event that BP readings fell within the hypertensive range, participants were advised to visit their General Practitioner for further investigation.

### Procedure

This study was approved by an institutional ethics review board and all participants gave informed consent. Saliva collection tubes and written instructions were posted to participants. The researcher then contacted participants by phone to explain the instructions, and to answer any queries. The study was carried out in the natural environment, as participants continued their typical daily routines. On the morning of the study, participants collected four saliva samples: immediately, 15, 30, and 45 min after waking (see Section 2.4). Participants then placed the completed samples into their domestic freezers. After the morning saliva samples had been collected, the researcher met with the participants (usually in their homes) and attached the ABP monitor (see Section 2.5). The researcher ensured that the initial reading was valid, and discussed the ABP monitoring with participants. Written instructions and contact details were also provided. Participants collected an additional saliva sample at 12:00.

ABP monitors were removed 24 h after they were attached. Participants were given a questionnaire booklet, which they completed and returned to the researcher by post.

### Salivary Analysis

Saliva samples were retrieved from participants, and stored frozen at -20̊C until they were assayed for cortisol and sAA in a bioassay laboratory. On the day of testing, samples were thawed, vortexed, and centrifuged at 3000 rpm for 15 min.

#### Cortisol

The four morning saliva samples were assayed in duplicate for salivary cortisol by enzyme immunoassay using the Salimetrics salivary cortisol assay kits (Salimetrics Europe, Suffolk, UK). The test requires 25 μL of saliva, and it has a range of sensitivity from .003–3.0 μg/dL. Further details on cortisol immunoassay can be obtained from Salimetrics (2012a). Average intra- and inter-assay coefficients of variability were less than 4 and 7%, respectively. The coefficient of variability refers to the standard deviation of a set of measurements divided by the mean of the set (Salimetrics 2013). Two area under the curve calculations were made: area under the curve with respect to ground (AUC_G_), and area under the curve with respect to increase (AUC_I_), using Pruessner et al.’s (2003) formulas (see Clow et al. 2004, for further information). Both the AUC_G_ (which represents overall cortisol secretory activity), and the AUC_I_ (a measure of the dynamic increase in cortisol secretion), have been found to be differentially associated with psychological health and wellbeing (Clow et al. 2004).

To control for noncompliance with saliva collection guidelines, samples were excluded if there was more than a 10-min difference between reports of waking and collecting the first sample. There was some additional loss of data due to some samples containing too little saliva for reliable analysis. After applying these criteria, sufficient data were available to include 23 parents from the ASD group (12 female and 11 male) and 24 parents from the control group (14 female and 10 male) in the cortisol (area under the curve) statistical analyses.

#### Alpha-Amylase

The 12:00 samples were assayed by kinetic measurement with the Salimetrics sAA assay kits (Salimetrics Europe, Suffolk, UK). The test used 10 μL of saliva. Further details on sAA assay can be obtained from Salimetrics (2012c). Average inter-assay coefficients of variability were less than 11%. To control for noncompliance with data collection guidelines, samples were excluded if they were not reported as being collected within 60 min of the target time (i.e., 12:00). Again, there was some additional loss of data due to some samples containing too little saliva for reliable analysis. After applying these exclusion criteria, sufficient data were available to include 22 parents from the ASD group (12 female and 10 male) and control group (11 male and 11 female) in subsequent statistical analyses.

### Data Analysis

Cortisol and sAA data were positively skewed, and were natural-log and square-root transformed, respectively (Adam and Kumari 2009; Granger et al. 2007). Outliers identified on boxplots were then recoded to the next highest value in the distribution (Tabachnick and Fidell 2001). Skewness statistics were less than 0.35 for all of the cortisol and sAA distributions after data transformation. For ease of interpretation, raw, untransformed values are presented in figures and tables.

Analysis of variance (ANOVA) tests were conducted to compare the ASD and control group with respect to each of the dependent variables. First, screening was conducted to determine whether any potential covariates were likely to influence the dependent variable. Pearson’s *r* correlations were conducted, and potential covariates that were not correlated with the dependent variable were excluded. Potential covariates that *were* correlated with the dependent variable were further screened to determine if they met the analysis of covariance (ANCOVA) assumptions of linearity, homogeneity of regression slopes (i.e., by customising the ANCOVA model to look at the independent variable x covariate interaction), and independence of covariates (i.e., Pearson’s *r* correlations were conducted between covariates to ensure they were not highly correlated with each other; Field 2005). ANCOVA analyses were then conducted for variables in which all of these assumptions had been met (i.e., anxiety, depression, sAA, and mean 24-h DBP and HR). For all other variables, ANOVA analyses were conducted. Alpha values were corrected using family-wise Bonferroni adjustment. For CV measures, results are presented for mean 24-h BP and HR analyses. Separate means for awake and asleep BP and HR measures were also examined (data not presented) but revealed no systematic differences.

## Results

### Descriptive Statistics

Mean scores for parent variables are presented in Table 2. Mean anxiety and depression scores fell within the normal range (0–7) for the control group (Zigmond and Snaith 1983). Mean depression scores for the ASD group also fell within the normal range, with mean anxiety scores falling within the mild range (8–10). However, anxiety scores for 13 parents (34%) from the ASD group and one parent from the control group fell within the moderate range (11–14), with anxiety scores for one parent from the ASD group, but no parents from the control group, falling within the severe range (15–21). Only two parents (5%) from the ASD group, but no parents from the control group, had depression scores within the moderate range, with no scores for either group falling within the severe range. Mean group parental distress scores fell at the 85th percentile for the ASD group, indicating clinically significant levels of parenting stress (Abidin 1995). Lower levels of parental distress were reported by parents in the control group, with mean group parental distress scores falling at the 25th percentile. Defensive responding scores for the ASD group (*M =* 19.21, *SD =* 5.31) and control group (*M* = 12.58, *SD* = 3.35) suggested that parents were not responding defensively to the PSI (scores of 10 or below are taken to indicate defensive responding).

**Table 2:** Mean scores for dependent variables for parents in the ASD (*N* = 38) and control (*N* = 38) groups

	ASD Group		Control Group	
Variable	M *(SD)*	Range	M *(SD)*	Range
Parenting responsibility	37.14 (10.20)	22–57	36.24 (9.71)	20–51
Perceived anxiety	8.29 (3.82)	0–16	4.68 (2.89)	0–11
Perceived depression	6.13 (3.14)	1–14	2.39 (1.84)	0–6
Parental distress	32.05 (8.40)	15–48	21.21 (5.60)	12–35
Cortisol (AUC_G_^a^)	-1.19 (0.46)	−2.06 – 0.04	−0.93 (0.45)	−1.63 – 0.06
Cortisol (AUC_I_^b^)	0.11 (0.61)	−0.75 – 1.36	0.20 (0.47)	−0.44 – 1.26
Cortisol 1	0.43 (0.36)	0.04–2.09	0.47 (0.20)	0.21–0.86
Cortisol 2	0.46 (0.33)	0.06–2.00	0.53 (0.21)	0.19–1.13
Cortisol 3	0.42 (0.21)	0.13–0.87	0.60 (0.23)	0.25–1.17
Cortisol 4	0.40 (19)	0.17–0.86	0.48 (0.20)	0.13–1.06
SAA^c^	144.96 (111.81)	8.20–390.20	100.20 (82.60)	16.10–261.80
Mean 24-h SBP^d^	129.28 (15.99)	99–168	130.67 (13.57)	107–162
Mean SBP^d^ night-time dip	14.05 (6.61)	0–24	15.10 (9.44)	−16 – 32
Mean 24-h DBP^e^	76.78 (11.27)	51–108	75.47 (8.08)	57–91
Mean DBP^e^ night-time dip	19.52 (7.75)	3–34	23.23 (8.91)	3–41
Mean 24-h HR^f^	74.03 (8.55)	60–94	70.42 (5.83)	61–84
Quantity reported illnesses	0.95 (1.35)	0–6	0.08 (0.27)	0–1

^a^AUC_G_: Area under the curve with respect to ground.

^b^AUC_I_: Area under the curve with respect to increase.

^c^sAA: Salivary alpha-amylase.

^d^SBP: Systolic blood pressure.

^e^DBP: Diastolic blood pressure.

^f^HR: Heart rate

**Fig. 1:**
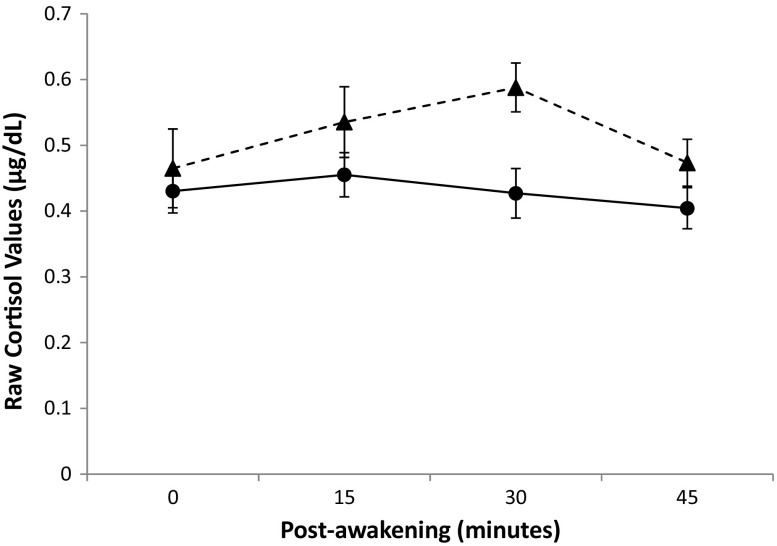
Mean values and standard errors bars for cortisol awakening responses (μg/dL) for parents in ASD (circles with solid line) and control (triangles with dashed line) groups

Mean cortisol and sAA levels for both groups are presented in Table 2, while mean cortisol values are also illustrated in Fig. 1. As can be seen in Table 2, mean 24-h SBP and DBP for both groups fell within the normotensive range, defined as a 24-h SBP ≤ 135 mmHg and a 24-h DBP ≤ 85 mmHg (O’Brien et al. 2000).

### Parent-Report Measures

Two-way ANOVA revealed a significant main effect for gender with respect to parenting responsibility, *F*(1,70) = 130.71, *p* < .001, partial η^2^ = .65, such that mothers reported higher levels of parenting responsibility than fathers. However, there was no significant main effect for group, and no significant group x gender interaction for parenting responsibility (*p* > .05). Two-way ANOVA conducted for parental distress revealed a significant main effect for group, *F*(1,72) = 44.65, *p* < .001, partial η^2^ = .38, with parents in the ASD group reporting significantly higher levels of parental distress than parents in the control group. However, no significant main effect for gender and no interaction effects were observed for parental distress (*p >* .05).

For anxiety and depression, two-way ANCOVA analyses were conducted with parenting responsibility included as a covariate. Bonferroni-adjusted ANCOVA analyses revealed significant main effects for group for both anxiety, *F*(1,69) = 32.51, *p* < .001, partial η^2^ = .32, and depression, *F*(1,69) = 51.27, *p* < .001, partial η^2^ = .43, with parents in the ASD group reporting significantly higher levels of anxiety and depression than parents in the control group. No significant main effects for gender and no significant group x gender interaction effects were observed for anxiety or depression (*p* > *.*025).

With respect to parent-reported quantity of illnesses, two-way ANOVA revealed significant main effects for group, *F*(1,72) = 17.17, *p* < .001, partial η^2^ = .19, and gender, *F*(1,72) = 6.95, *p* = .01, partial η^2^ = .09, such that mothers reported a higher number of illnesses than fathers, and participants in the ASD group reported a higher number of illnesses than participants in the control group. A significant interaction effect for group x gender was also observed, *F*(1,72) = 5.69, *p* = .02, partial η^2^ = .07, with mothers of children with ASD reporting the highest number of illnesses.

### Salivary Biomarkers

Two-way ANOVA with Bonferroni correction revealed that there were no significant main or interaction effects for AUC_G_ or AUC_I_ (*p* > .025). Analyses were also conducted to compare group differences on individual cortisol measures. Bonferroni-adjusted two-way ANOVA analyses of individual cortisol measures revealed a significant main effect for group for the third cortisol measure, *F*(1,56) = 11.66, *p* = .001, partial η^2^ = .17, such that parents in the ASD group had significantly lower cortisol levels on the third morning measure (i.e., 30 min after waking) than parents in the control group. No other significant effects were observed for the individual cortisol measures (*p >* .013). Two-way ANCOVA with participant age included as a covariate found no significant main or interaction effects for sAA (*p >* .05). Results for AUC_G_, AUC_I_, and sAA measures did not differ when controlling for sleep measures (i.e., parent-reported sleep quality as measured by the PSQI, and the reported duration of sleep on the night before sample collection).

### CV Activity

Two-way ANOVA with Bonferroni adjustment revealed a significant main effect for gender for 24-h SBP, *F*(1,76) = 11.44, *p* = .011, partial η^2^ = .14, with fathers found to have significantly higher mean 24-h SBP than mothers across both parenting groups. No other significant effects were observed for mean 24-h SBP or SBP dip (*p >* .025). A similar pattern emerged with the DBP measures (i.e., 24-h DBP and DBP dip). Bonferroni-adjusted two-way ANCOVA, with body mass index (BMI) included as a covariate, revealed significant main effects for gender for 24-h DBP, *F*(1,62) = 9.19, *p* = .004, partial η^2^ = .14, with fathers found to have significantly higher mean 24-h DBP than mothers irrespective of parenting group. No other significant effects were found for mean DBP measures (*p >* .025). Similarly, ANCOVA with BMI included as a covariate revealed a significant main effect for gender for mean 24-h HR, *F*(1,59) = 8.45, *p* = .005, partial η^2^ = .13. However, in contrast to the significant gender effects for SBP and DBP, mothers had significantly higher mean 24-h HR than fathers. No other significant differences were found for mean 24-h HR (*p >* .05).

In order to determine whether use of antihypertensive medication influenced the findings in relation to CV measures, all analyses that included CV measures were repeated excluding any participant taking antihypertensive medication. The outcomes of the analyses did not differ when these participants were excluded, so it was determined that these participants’ data were not skewing the findings and they were retained in the CV analyses. Similar to cortisol and sAA analyses, results did not differ when controlling for sleep measures (i.e., parent-reported sleep quality and duration of sleep on the night of data collection).

### Correlation between Self-Report and Physiological Measures

Pearson correlations were conducted between parent-reported measures (i.e., parental distress, anxiety, and depression), physiological measures (i.e., AUC_G_, AUC_I_, and sAA), and other health-relevant variables (i.e., parental responsibility, parent-reported sleep quality, participant age, and the number of reported illnesses). Results are presented in Table 3. Significant positive correlations were found between all parent-reported measures (i.e., parental distress, anxiety, depression, and sleep quality; *p* < .05). However, no significant correlations were found between the parent-reported measures and the physiological measures (*p* > .05). The number of parent-reported illnesses was positively correlated with parent-reported measures of wellbeing (i.e., parental distress, *r* = .37, *p* < .001; anxiety, *r =* .40, *p* < .01; and depression, *r* = .43, *p* < .001) and sleep quality, *r* = .45, *p* < .001, but was not significantly correlated with any of the physiological measures (*p* > .05). The only other significant correlations were between participant age and sAA (*r* = .36, *p* < .05), and between 24-h DBP and both SBP night-time reduction (*r =* .85, *p* < .001) and DBP night-time reduction (*r =* .78, *p <* .001).

**Table 3:** Pearson correlations between parent-report and physiological measures for all participants (*N =* 76)

Measure	1	2	3	4	5	6	7	8	9	10	11	12	13	14	15
1. Parental distress (PSI^a^)		.75**	.60**	.27*	.38**	.17	.37*	−.05	.04	−.01	.10	−.22	.10	−.26	.24
2. Anxiety (HADS^b^)			.76**	.33*	.42**	.13	.40*	−.01	−.10	−.21	−.24	−.18	−.20	−.26	.04
3. Depression (HADS^b^)				.22	.36**	.24	.43**	.02	.03	−.14	−.08	−.26	−.04	−.27	.10
4. Parental responsibility (PRS^c^)					−.05	−.04	.27	.14	−.14	−.16	−.10	−.24	−.25	−.14	−.02
5. Sleep quality (PSQI^d^)						−.04	.45**	.07	.04	−.07	−.09	−.01	.01	−.01	−.04
6. Participant age							.24	−.15	.03	.36*	−.03	−.13	.17	−.08	.12
7. Number of reported illnesses								−.14	.20	.10	.03	−.13	.03	−.15	.25
8. AUC_G_^e^									.35*	−.16	.17	−.03	−.02	.31	−.31
9. AUC_I_^f^										.18	.16	−.04	.06	.05	−.11
10. Alpha-amylase											.11	−.01	.19	−.04	.08
11. Mean 24-h SBP^g^												−.02	.85**	.13	.12
12. SBP^g^% Dip (Night-time)													−.03	.78**	.03
13. Mean 24-h DBP^h^														.04	.28*
14. DBP^h^% Dip (Night-time)															−.07
15. Mean 24-h HR^i^															

^a^PSI: Parenting Stress Index (Abidin 1995).

^b^HADS: Hospital Anxiety and Depression Scales (Zigmond and Snaith 1983).

^c^PRS: Parenting Responsibility Scale (McBride and Mills 1993).

^d^PSQI: Pittsburgh Sleep Quality Index (Buysse et al. 1989).

^e^AUC^G^: Area under the curve with respect to ground.

^f^AUC_I_: Area under the curve with respect to increase.

^g^SBP: Systolic blood pressure.

^h^DBP: Diastolic blood pressure.

^i^HR: Heart rate

### Coping Strategies and Social Support

Between-group ANOVAs were also conducted to compare coping strategies and social support between the two groups. Significant main effects for group, *F*(1,32) = 6.76, *p* = .01, partial η^2^ = .17, and gender, *F*(1,32) = 5.64, *p* = .02, partial η^2^ = .15, were found for emotional support, while main effects for group, *F*(1,32) = 12.90, *p* = .001, partial η^2^ = .29, and gender, *F*(1,32) = 4.65, *p =* .04, partial η^2^ = = .13, were also found for positive reframing, such that parents of children with ASD and mothers reported higher use of these coping strategies. Furthermore, significant main effects for group were found for planning, *F*(1,32) = 7.95, *p =* .01, partial η^2^ = .20, humour, *F*(1,32) = 8.60, *p =* .01, partial η^2^ = .21, acceptance, *F*(1,32) = 11.59, *p =* .002, partial η^2^ = .27, and religion, *F*(1,32) = 4.30, *p =* .04, partial η^2^ = .12, such that parents of children with ASD reported significantly higher use of these coping strategies than parents of children with typical development. No other significant group, gender or interaction effects were found (*p* > .05) for the remaining coping strategies (i.e., active coping, denial, substance use, instrumental support, behavioural disengagement, and self-blame). Furthermore, no significant group, gender, nor interaction effects were found for social support quantity or quality (*p* > .05).

## Discussion

The present study compared parent-report and selected physiological measures of stress between parents of children with and without ASD, and between mothers and fathers, in addition to testing the interaction effect between parenting group and gender. Mothers reported higher parenting responsibility than fathers across both parenting groups. Parents of children with ASD reported significantly higher levels of parental distress, anxiety, and depression than parents of typically developing children, with no significant gender differences observed. Parents of children with ASD also had lower levels of cortisol on the third morning cortisol measure, but the parenting groups did not differ significantly with respect to other cortisol measures, sAA levels, or CV activity, and no significant differences were observed between mothers and fathers. Significant correlations were found between all of the self-reported measures (i.e., parental distress, anxiety, and depression), whereas these variables were not correlated with the physiological measures. Additionally, while parent-reported illnesses correlated with parent-reported distress, anxiety, and depression, reported illnesses were not correlated with any of the physiological measures. Parents of children with ASD reported significantly higher use of a number of coping strategies (i.e., emotional support, positive reframing, planning, humour, acceptance, and religion) than parents of typically developing children.

Levels of parenting responsibility did not differ significantly between the two parenting groups, but mothers within both parenting groups reported significantly higher levels of parenting responsibility than fathers. This is consistent with previous research on paternal involvement (Lamb and Tamis-Lemonda 2004), indicating that mothers continue to be the primary caregivers in many families. Regardless of gender, parents of children with ASD reported significantly higher levels of anxiety, depression, and parental distress than parents of typically developing children. These results are consistent with previous findings that parents of children with ASD report higher psychological distress than parents of typically developing children (e.g., Duarte et al. 2005; Hayes and Watson 2013; Rao and Beidel 2009). Furthermore, average parental distress scores for parents of children with ASD in the present study were in the clinically significant range, suggesting unmet need for stress-reduction interventions for this population. Additionally, given the ease of completing the PSI-SF, routine screening could identify parents who are most in need. For instance, incorporating the PSI-SF into health checks, diagnostic assessments, or intervention reviews could identify parents experiencing clinically significant distress.

Previous literature has indicated that mothers and fathers of children with ASD may be at risk of experiencing blunted cortisol responses (e.g., De Andres-Garcia et al. 2012; Seltzer et al. 2010). In the present study, AUC_G_, AUC_I,_ and sAA levels did not differ significantly between the two parenting groups, or between mothers and fathers. However, analysis of absolute cortisol values revealed that parents of children with ASD had significantly lower levels of cortisol on the third awakening response (i.e., 30 min after waking) than parents of typically developing children, regardless of gender. Cortisol levels, which typically reach their peak 30 min after waking, were at their highest for parents of typically developing children for this measure, but had actually declined from baseline for parents of children with ASD, suggesting possible dysregulation to cortisol awakening responses among parents of children with ASD. Given the potential links between blunted cortisol activity and stress-related diseases (Heim et al. 2000), these tentative findings warrant further investigation. With respect to CV activity, Gallagher and Whiteley (2012) reported that caregivers of children with developmental disabilities, including ASD, had higher mean SBP than caregivers of typically developing children. In the present study, significant gender differences in BP and HR were observed, which is unsurprising given that men are known to experience higher BP and lower HR than women (Fuster and Kelly 2010). However, unlike Gallagher and Whiteley (2012), CV activity in the present study did not differ significantly between the two parenting groups, despite the fact that the parents of children with ASD reported significantly higher levels of distress, anxiety, and depression.

Further analyses revealed that while parent reports of distress, anxiety, and depression were correlated with one another, they were not significantly correlated with any of the physiological measures. These analyses also found that parent-reported illnesses, while correlated with parent-reported stress, anxiety, and depression, were not correlated with cortisol, sAA, or CV measures. The fact that the various parental reports were correlated with one another, but not with any physiological measures, warrants further consideration. As discussed earlier, much of the existing literature regarding stress and health among parents of children with ASD relies exclusively on parent reports of stress, health, and illness (e.g., Allik et al. 2006; Mugno et al. 2007). That parent-report measures did not correlate with physiological measures in the present study indicates that caution may be needed in relation to the use and interpretation of self-reported stress and health in research on wellbeing among parents of children with ASD, as well as stress research more generally. There are issues of potential bias in self-report measures (e.g., respondents’ perceptions about how they should respond could result in over- or under-reporting of symptoms). However, it is also possible that despite parents of children with ASD encountering stressors, certain protective factors may mediate the impact of those stressors on their physiological functioning.

It is known that social support and coping strategies have potential to moderate the impact of stressors on outcomes of parents of children with ASD (e.g., Boyd 2002; Dunn et al. 2001; Hastings et al. 2005). While no differences were found between the two parenting groups or between mothers and fathers in relation to quality or quantity of social support, significant group differences were found in relation to the use of a number of coping strategies. Parents of children with ASD reported higher use of emotional support, positive reframing, planning, humour, acceptance, and religion than parents of typically-developing children. These are primarily considered to be adaptive, positive coping strategies, and the fact that parents of children with ASD reported higher use of these strategies offers a possible explanation of the general absence of between-group differences in physiological responses. Higher use of adaptive coping strategies, including emotional support, by parents of children with ASD could act as a buffer to the negative impact of parenting stress, suggesting a health-protective effect of these adaptive coping strategies.

Reports of lower cortisol levels in previous literature for some parents of children with ASD received partial support in the present study. Such physiological adaptations are hypothesised to serve a protective function for individuals who experience chronic stress (Fries et al. 2005). For example, a blunted cortisol awakening response may offer some protection against potential health-damaging effects from cortisol rendered persistently high due to exposure to chronically stressful circumstances. The complex nature of such processes, along with the relative dearth of relevant published studies, suggests the need for additional research to further elucidate potential health-related threats associated with caring for a child with ASD. Investigation of factors and processes that may lead to positive adaptation and coping is also warranted. Research is needed to inform interventions and supports for parents of children with ASD and other disabilities, while simultaneously advancing general understanding of the mechanisms involved in chronic stress and adaptation.

While the present study highlights the importance of incorporating physiological measures into parental stress research, there are a number of factors that need to be considered when interpreting the results. In the present study, extraneous life-events or stressors, such as bereavement or a recent job loss, were not controlled for in either group. Any such confounds, were they present, are likely to have been exacerbated by the relatively small sample size, which also would have contributed to a lessening of statistical power in the study overall. However, as the first study to directly compare parent-reported stress, anxiety, depression, salivary biomarkers and 24-h ambulatory BP between parents of children with ASD and typically-developing children, this study has potential to guide future research in this area. The suggestion of a possible influence of adaptive and protective mechanisms in the present study highlights the importance of directly measuring physiological markers of stress rather than relying on self-report measures alone. It is important that such factors are comprehensively understood in order that the health needs of parents of children with ASD are supported by interventions (e.g., health screening, stress-reduction interventions, and support in developing adaptive coping strategies) that best meet their specific needs.
